# Biological and environmental influences on motor coordination in Peruvian children and adolescents

**DOI:** 10.1038/s41598-021-95075-7

**Published:** 2021-07-29

**Authors:** Sara Pereira, Alcibíades Bustamante, Carla Santos, Donald Hedeker, Go Tani, Rui Garganta, Olga Vasconcelos, Adam Baxter-Jones, Peter T. Katzmarzyk, José Maia

**Affiliations:** 1 CIFI2D, Faculty of Sport, University of Porto, R. Dr. Plácido da Costa 91, 4200-450 Porto, Portugal; 2 CIDEFES, Lusófona University, Lisbon, Portugal; 3 School of Physical Education and Sports, National University of Education Enrique Guzmán y Valle, La Cantuta, Lurigancho-Chosica, Peru; 4 Department of Public Health Sciences, University of Chicago, Chicago, IL USA; 5 School of Physical Education and Sports, University of São Paulo, São Paulo, Brazil; 6 College of Kinesiology, University of Saskatchewan, Saskatoon, Canada; 7 Pennington Biomedical Research Center, Louisiana State University, Baton Rouge, LA USA

**Keywords:** Epidemiology, Risk factors

## Abstract

This study investigated the associations between biological and environmental factors and gross-motor coordination (GMC) in Peruvian children and adolescents. The sample comprised 7401 boys and girls, aged 6–14 years, recruited from three geographical regions: sea-level, Amazon and high-altitude. Biological variables included age, sex, height, BMI, physical fitness, stunting, and maturational status. Environmental influences included geographical region and school characteristics. Gross-motor coordination was tested with the *Körperkoordinationstest für Kinder* and the data analyzed by multilevel logistic regression. Results showed a high prevalence of below normal GMC scores. Sex, age, geographical area, biological maturation, BMI (normal versus overweight/obesity), and stunting were all significant predictors of GMC. There was also an interaction between age, sex, and geographical area indicating that older girls who lived at sea-level and high-altitude were more likely to display below normal GMC scores. The school context was less important in predicting GMC problems than the interplay between biological characteristics and geographical region. These results suggest that early identification, as well as educational and pediatric care interventions, are of importance in reducing below normal GMC among Peruvian children and adolescents.

## Introduction

The description and interpretation of children and adolescents’ physical status are of great importance^1^ given the known relationship with their health and well-being^2^. Moreover, healthy children and adolescents are more likely to succeed in school^3^, be physically active and more physically fit^4^, as well as have better motor coordination^5^.

Gross motor coordination (GMC) is defined as the harmonious and economical interaction of the musculoskeletal, nervous, and sensory systems that produce accurate and balanced motoric actions with minimal energy expenditure^6^. Children with below normal GMC scores typically experience difficulties in performing motor tasks. They also face challenges related to their daily activities, school performance and social participation^7–9^. The prevalence of GMC problems/disorders ranges from about 9- to 28% in children and adolescents^10–12^ and appears to be higher in countries with lower human development indexes^13^. Most experts agree that below normal GMC scores need to be addressed as early as possible to prevent extension into adulthood. If left unresolved these childhood GMC issues could interfere with the demands of future work and daily living activities^14^.

It has been reported that GMC levels vary substantially among children and adolescents, and that individual demographics, biological variables (e.g., age, sex, body mass index, physical fitness, gestational age), socioeconomic status, parental education, number of siblings, or birth order and environmental characteristics (e.g., time spent outdoors in playing spaces, parents’ physical activity and family interactions), are modulating factors^15–17^. Available reports regarding the role of environmental factors in explaining GMC variation are limited and controversial^12, 18, 19^. Therefore, new studies are needed to better understand these linkages. For example, Barnett et al.^11^ showed inconsistent results regarding the influence of socioeconomic status on GMC. These findings were like those of Niemistö et al.^19^ who studied children from three geographical regions of Finland, scattered by residential density areas. Previous studies have rarely explored the impact of putative covariates that influence children’s chances of having below normal GMC, and instead mostly concentrated on the associations with physical fitness and body composition^12, 20–22^.

Another issue of interest is geographical location (e.g., land elevation levels), a possible marker of a child’s environmental exposure. Environmental differences within countries may play an important role in shaping motor and GMC development and thus needs to be considered^23^. Peru can be described in terms of three broad regions: the arid Coastal region in the west at sea level, the Sierra central region at high-altitude and the forested Amazonia in the east. Systematic differences in children and adolescents´ lifestyles in these three regions are well known, as they have vastly different daily chores and subsistence activities^24^. All of these environmental issues are known to exert different influences on an individual’s growth and development^25^. For example, de Chaves et al.^12^ reported that Peruvian children and adolescents living at sea level or at high-altitude were more likely to suffer from GMC difficulties than those living in the Amazon region.

School characteristics are also important and commonly examined correlates with respect to GMC. Children and adolescents spend a large portion of their waking hours at school^26^, and it is expected that school characteristics play an important role in addressing and resolving below normal GMC scores. For example, Santos et al.^23^ reported that in Peruvian children, 31% of the total variance in GMC (based on continuous raw scores) was explained by school characteristics, namely school size, availability of playgrounds with obstacles, whether there was an indoor multi-sport complex, and if physical education classes were of longer duration. It is likely that there is an interaction between the natural and built environments, and individual characteristics on GMC but this is seldom tested. To our knowledge, there is a lack of information on the interplay between GMC and a wide range of biological and environmental factors; this is especially with respect to those identified as performing below normal.

To better understand this integrative questioning, an appropriate analytical framework is required. Using a multilevel statistical model, which accounts for the nested structure of the data, it is possible to probe the network of independent and interaction links between biological and environmental characteristics and GMC within and between individuals. Specifically, the aims of this study were to expand the previous work of our research group^12, 27^ and address the following questions: (1) Are age, sex and geographical areas associated with children and adolescents’ below normal GMC during childhood and adolescence? (2) Do age-by-sex, age-by-geographical area, and sex-by-geographical area interactions predict below normal GMC? (3) Do age-by-sex-by-geographical area interactions predict below normal GMC? (4) What are the impacts of child-level characteristics on the likelihood of below normal GMC? And, (5) Do school-level variables influence below normal GMC? We hypothesized that age, sex, child and school level characteristic’s, geographical regions and their interactions would be associated with below normal GMC.

## Results

### Descriptive statistics

Table 1 shows the child-level characteristics by GMC level (normal and below normal). An increase in the prevalence of below normal GMC scores from 6–10 years to 11–14 years was observed (χ^2^ = 216.035, p < 0.05). Girls (50.6%) had higher prevalence of below normal GMC scores compared to boys (18.3%, χ^2^ = 824.330, p < 0.05). Children and adolescents from sea-level (49.0%) and high-altitude (45.4%) had a higher prevalence of below normal GMC scores compared to their Amazonian peers (27.5%). Moreover, children and adolescents with stunting had a higher prevalence than non-stunted children and adolescents (40.0% vs 5.8%, χ^2^ = 7.150, p < 0.05). A similar result was observed regarding weight status in which youth who were overweight or obese had a higher prevalence of below normal GMC scores than their normal weight peers (40.0% vs 34.8%, χ^2^ = 17.875, p < 0.05). Finally, those with high levels of physical fitness had a lower prevalence of GMC problems (22.7%) when compared with those showing low (48.4%) and medium (37.0%) levels of fitness.

**Table 1:** Child-level characteristics by gross motor coordination.

		Gross motor coordination			
		Normal	Below normal	χ^2^	Comparisons^a^
		N (%)	N (%)		
Age class	6–10 years	2647 (72.0)	1030 (28.0)	216.035***	11–14 years > 6–10 years
	11–14 years	2069 (55.6)	1655 (44.4)		
Sex	Girls	2036 (49.4)	2085 (50.6)	824.334***	Girls > boys
	Boys	2680 (81.7)	600 (18.3)		
Geographical area	Sea-level	749 (51.0)	719 (49.0)	305.716***	S > A H > A
	High-altitude	1017 (54.6)	847 (45.4)		
	Amazon region	2950 (72.5)	1119 (27.5)		
Stunting	Non-stunted	4269 (64.2)	2378 (35.8)	7.150**	Stunted > non-stunted
	Stunted	447 (59.3)	307 (40.7)		
Body Mass Index	Normal weight	3416 (65.2)	1820 (34.8)	17.875***	OW/O > NW
	Overweight/obese	1300 (60.0)	865 (40.0)		
Physical fitness	Low	950 (51.6)	892 (48.4)	266.569***	L > M > H
	Medium	2339 (63.0)	1375 (37.0)		
	High	1427 (77.3)	418 (22.7)		

^a^Direction of association for below normal GMC scores.

**p<0.01; ***p<0.001.

School-level characteristics are shown in Table 2. Eighty-three percent of schools were located in urban areas, with the number of students per school ranging from 96 to 1200. Furthermore, 83.3% of schools had a playground without obstacles, and 33.3% had access to an indoor multi-sport complex. Forty-four percent of schools had neither policies nor practices for physical activity, whereas 16.7% had policies and 38.9% had practices for physical activity. In 55.6% of schools, physical education classes were more than 90 min in duration once a week, and children and adolescents active time during classes was, on average, 78 min. Additionally, 77.8% of schools offered extracurricular activities, and 38.9% allowed students to use school infrastructures outside of school activities.

**Table 2:** Descriptive statistics of the school-level characteristics [counts (n), frequencies (%), means, standard deviations (SD) and ranges].

	n	%
**School setting**		
Mixed	3	16.7
Urban	15	83.3
**Policies and practices for physical activity**		
No	8	44.4
Only policies	3	16.7
Only practices	7	38.7
**Playground obstacles**		
Without obstacles	15	83.3
With obstacles	3	16.7
**Indoor multi-sport complex**		
No	12	66.7
Yes	6	33.3
**Physical education classes duration**		
≤ 90 min	8	44.4
> 90 min	10	55.6
**Extracurricular activities**		
No	4	22.2
Yes	14	77.8
**School infrastructures available outside of school activities**		
No	11	61.1
Yes	7	38.9

	Mean (SD)	Range
Number of students per school	453 (263)	96–1200
Active time during physical education class	78 (16)	50–110

Results of the multilevel logistic analysis are presented in Table 3. The null model (data not shown) indicated that 15% of the total variance in GMC categories was explained by school-level characteristics, with the remaining 85% associated with distinct child and adolescent traits.

**Table 3:** Parameters estimates [odds ratios (OR) and 95% confidence intervals] and variance components for gross motor coordination.

Variables	Model 1	Model 2	Model 3	Model 4	Model 5
	OR (95%CI)	OR (95%CI)	OR (95%CI)	OR (95%CI)	OR (95%CI)
Age (11–14 years)	1.686 (1.454–1.956)***	1.621 (1.217–2.159)**	1.268 (0.919–1.749)	0.556 (0.385–0.801)**	0.582 (0.406–0.835)**
Sex (boys)	0.183 (0.161–0.209)***	0.338 (0.198–0.578)***	0.221 (0.119–0.409)***	0.126 (0.064–0.247)***	0.077 (0.037–0.160)***
Geographical area (high-altitude)^e^	0.904 (0.554–1.476)	1.263 (0.709–2.246)	1.239 (0.669–2.293)	1.514 (0.740–3.098)	1.505 (0.369–6.148)
Geographical area (amazon region)^e^	0.366 (0.230–0.581)***	0.360 (0.212–0.614)*	0.319 (0.181–0.561)***	0.425 (0.220–0.821)*	0.711 (0.279–1.811)
**Interactions (two-way)**					
Age-by-sex^c^		0.722 (0.564–0.925)*	1.918 (1.008–3.651)*	0.872 (0.438–1.737)	0.703 (0.361–1.367)
Age-by-geographical area (11–14 years/high-altitude)		0.886 (0.591–1.330)	1.057 (0.662–1.690)	0.854 (0.520–1.403)	0.825 (0.496–1.372)
Age-by-geographical area (11–14 years/amazon-region)		1.450 (1.019–2.063)*	2.131(1.420–3.197)***	2.428 (1.570–3.756)***	2.406 (1.575–3.674)***
Sex-by-geographical area (male/high-altitude)		0.560 (0.319–0.984)*	0.786 (0.395–1.565)	0.578 (0.275–1.212)	0.947 (0.427–2.100)
Sex-by-geographical area (male/amazon region)		0.673 (0.387–1.170)	1.252 (0.395–1.565)	1.120 (0.550–2.282)	1.838 (0.848–3.980)
**Interactions (three-way)**					
Age-by-sex-by-geographical area (11–14 years/male/high-altitude)			0.439 (0.205–0.944)*	0.512 (0.229–1.142)	0.633 (0.289–1.386)
Age-by-sex-by-geographical area (11–14 years/male/amazon region)			0.247 (0.119–0.514)***	0.269 (0.124–0.581)**	0.325 (0.154–0.687)**
**Other child-level characteristics**					
Maturity offset				1.820 (1.651–2.005)***	1.823 (1.652–2.011)***
Stunting (yes)^b^				1.491 (1.232–1.804)***	1.484 (1.227–1.795)***
Body mass index^d^ (overweight/obese)				1.291 (1.131–1.473)***	1.284 (1.125–1.465)***
Physical fitness (medium)				0.419 (0.361–0.487)***	0.419 (0.360–0.486)***
Physical fitness (high)				0.150 (0.122–0.185)***	0.150 (0.122–0.184)***
**School-level characteristics**					
School setting (urban)^f^					1.030 (0.487–2.178)
Number of students^g^					0.999 (0.998–1.000)
Policies or practices for physical activity (only policies)^h^					3.197 (1.014–10.080)*
Policies or practices for physical activity (only practices)^i^					0.948 (0.504–1.783)
Playground obstacles (with obstacles)^j^					1.597 (0.881–2.897)
Indoor multisports (yes)^j^					1.551 (0.730–3.296)
Physical education classes duration (≥ 90 min)^l^					0.979 (0.586–1.638)
Active time during physical education classes					1.010 (0.992–1.027)
Extracurricular activities (yes)^j^					0.525 (0.279–0.987)*
Deviance	8210.68	8189.64	8174.48	7740.20	7729.12
Number of parameters	6	11	13	18	27
∆ in Deviance from previous model (∆ in number of parameters)	775.15 (4)***	21.04 (5)***	15.16 (2)***	434.28 (5)***	11.08 (9)

***p < 0.001;**p < 0.01; *p < 0.05; ^a^normal growth is the reference; ^b^girls are the reference; ^c^thiness/normal weight is the reference; ^d^Amazon region is the reference; ^e^mixed is the reference; ^f^number of students divided by 10; ^g^no policies nor practices is the reference; ^h^without obstacles is the reference; ^i^no is the reference; ^j^ < 90 min is the reference.

Question 1: Are age, sex and geographical area associated with children and adolescents’ chances of having below normal GMC scores? Model 1 results show that older individuals were more likely to exhibit below normal GMC scores than their younger peers (OR = 1.686; 95%CI 1.454–1.956). Moreover, boys (OR = 0.183; 95%CI 0.161–0.209) and those living in the Amazon region (OR = 0.366; 95%CI 0.230–0.581) were less likely to show below normal GMC scores than girls and their sea-level peers, respectively.

**Figure 1:**
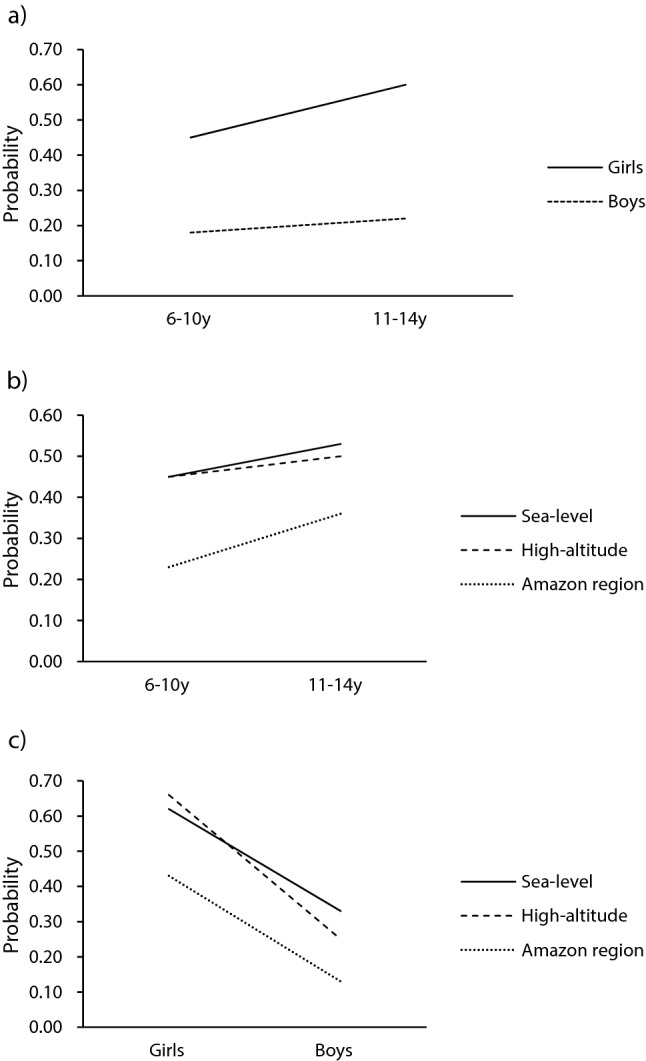
Plots of probabilities of having below normal GMC scores: age-by-sex (**a**) age-by-geographical location (**b**) and sex-by-geographical location (**c**) interactions.

Question 2: Do age-by-sex, age-by-geographical area, and sex-by-geographical area interactions condition below normal GMC scores? Model 2 was a better fit of the data than Model 1 (χ^2^ = 21.04, 5 df, p < 0.001) as indicated by a significant drop in the deviance statistic. The model also found that interaction effects had statistically independent effects. The age-by-sex interaction results revealed that although both boys and girls increased the probability of having below normal GMC scores from 6–10 years through to 11–14 years, the increase with age was greatest in girls (see Fig. 1a). For the age-by-geographical area interaction, a significant effect (p < 0.05) was only observed between sea-level and amazon regions, indicating that sea-level youth have a greater chance of having below normal GMC scores. Figure 1b shows a lower probability for Amazonian subjects in both age classes (6–10 years = 23% and 11–14 years = 36%) to demonstrate below normal GMC scores when compared to sea-level peers (6–10 years = 45% and 11–14 years = 53%) and high-altitude peers (6–10 years = 45% and 11–14 years = 50%). However, it is important to note that the difference in rates of below normal GMC scores for Amazonian subjects tend to be higher (~ 14%) than their counterparts from sea-level (~ 8%) and high-altitude (~ 5%) across age. Finally, there was a significant sex-by-geographical area interaction (Fig. 1c), but only between sea-level and high-altitude Peruvian youth of both sexes.

**Figure 2:**
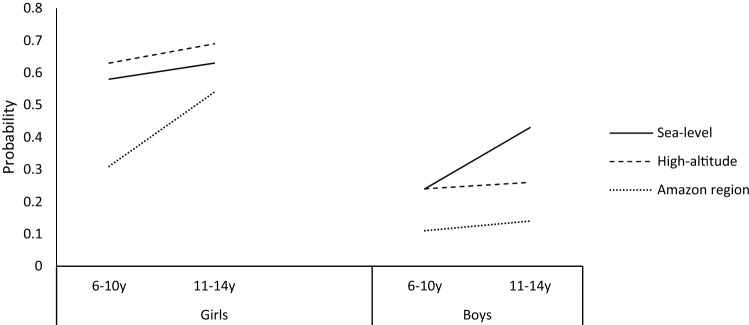
Plots of probabilities of having below normal GMC scores: age-by-sex-by-geographical area interaction.

Question 3: If the two-way interactions are significant, does an age-by-sex-by-geographical area interaction predict below normal GMC scores? Model 3 was a better fit of the data than Model 2 (χ^2^ = 15.16, 2 df, p < 0.001) indicating the importance of three-way interactions in both categories. Figure 2 shows that girls from the Amazon region had higher increases of the probability of having below normal GMC scores between 6 and 10 years through to 11–14 years (23%) than sea-level (5%) and high-altitude (3%) peers. Boys showed a different pattern: at 6–10 years boys from sea-level and high-altitude had equal probability of having below normal GMC scores (24%) and higher probability than Amazon region (11%). Additionally, boys from high-altitude and Amazon region from 6–10 years to 11–14 years displayed residual increases in their probability of having below normal GMC scores (2% and 3%, respectively), although boys from the sea-level presented higher increases in their probability (19%).

Question 4: What are the relationships of child-level characteristics on the likelihood of below normal GMC scores? Model 4 was a better fit of the data than Model 3 (χ^2^ = 434.28, 5 df, p < 0.001), and showed that those advanced in their biological maturation (OR = 1.820; 95%CI 1.651–2.005), stunted (OR = 1.491; 95%CI 1.232–1.804), and being overweight or obese (OR = 1.291; 95%CI 1.131–1.473) were more likely to have below normal GMC scores. However, Peruvians with medium (OR = 0.419; 95%CI 0.361–0.487) and higher (OR = 0.150; 95%CI 0.122–0.185) physical fitness levels had lower odds ratios, i.e., they were more protected of displaying below normal GMC scores than their peers with low physical fitness levels.

Question 5: Do school level variables influence below normal GMC scores? Finally, when school-level covariates were added (Model 5), no improvement in model fit was found relative to Model 4 (χ^2^ = 11.08, 9 df, p > 0.05). This suggests that, after accounting for individual characteristics and geographical location as well as their significant interactions, school-level characteristics did not significantly predict Peruvian children and adolescents’ chances of having below normal GMC scores.

## Discussion

There is little doubt that identifying children with below normal GMC scores, and their putative correlates, during a child’s growth has important long term educational and pediatric care implications^28^. We believe that the uniqueness of the present study is in the examination of the interactions between individual and environmental characteristics in explaining the presence of below normal GMC scores during childhood. This is of particular importance for children living in developing countries, such as Peru, but can also be extended to other South-American children living in regions marked by geographical, cultural, and socioeconomic differences.

An important finding of this study is that the main fraction of GMC categories’ variance (85%) was explained by Peruvians’ individual characteristics, and this is similar to a report with Portuguese children^18^. Also consistent with previous results is the fact that Peruvian girls are more likely to display below normal GMC scores than Peruvian boys^12, 27^. This sex difference in GMC suggests a complex interplay between biological and cultural factors, which are probably linked to differences in sport participation, daily chores and the different physical activities chosen by girls compared to boys. For example, Peruvian girls tend to spend their time after school in home activities (e.g., cleaning, cooking, etc.) whereas boys spend their time in sports’ participation and other varied physical activities^29^.

We showed that older Peruvians were more likely to have below normal GMC scores, a result similar to a finding in Flemish children aged 5–12 years^30^. One plausible explanation for such results may be linked to differences in the timing and tempo of the adolescent growth spurt. This period of growth is marked by an asynchrony in the growth of different parts of the body^31, 32^ that can adversely affect motor control, a phenomenon termed “clumsiness” or ‘‘adolescent awkwardness’’^33^. It is also possible that interindividual differences, within the same sex, in biological maturation timing and tempo may explain our results.

For example, Bisi and Stagni^34^ in an experimental study compared the gait performance of fast growing (height increase > 3 cm in 3 months) and slow growing (height increase < 1 cm in 3 months) male adolescents. It was found that during a walking task the association with the timing of the adolescent growth spurt influenced gait variability, smoothness, and regularity. Additionally, whereas the slow and fast-growing children performed some motor tasks awkwardly, those who grew steadily were able to cope with their physical growth changes and thus maintained smoothness and regularity in some motor tasks. This result was also previously reported in Flemish male soccer players^35^ as well as in healthy Belgium boys^36^. Indeed, these studies showed a temporary decline in performance during the growth spurt. Further, Loko et al.^37^ working with Estonian adolescent girls, reported plateaus and declines in multiple motor skills’ performance around the adolescent growth spurt. In spite of these findings, more longitudinal studies are still required to better understand the potential associated mechanisms^38^, namely sensorimotor function and sex differences^38, 39^. More specifically, boys and girls experience puberty at different timing^23^ and at this time the sensorimotor mechanisms are not fully mature such as neurocognitive processing capabilities, neuromuscular control and coordination, and regulation of postural control^38^. These issues allied to the rapid growth spurt may help explain the higher prevalence of below normal GMC in older Peruvians.

The main challenge of the present study was to investigate how geographical areas interacted with individual-level characteristics (age and sex) in predicting below normal GMC scores. Based on a three-way interaction of age-by-sex-by-geographical area we showed that older girls living at sea level were more likely to display below normal GMC scores, whilst the youngest boys from the Amazonian region were less likely to have below normal GMC scores. These results emphasize the importance of identifying differences between these three regions that are contributing to different probabilities of below normal GMC scores, especially between the sea level and the Amazon regions. One possible explanation could be differences in their natural, sociodemographic, health care and cultural characteristics (see Table 4) that influence lifestyle behaviors and routine activities. For example, the sea-level region has a higher human developmental index, higher income and consequently a better urban development and higher population density. In such environments children tend to adopt certain lifestyle behaviors, and other daily routine activities, closer to youth from developed countries i.e., more sedentary activities and lower levels of physical activity. In fact, Sharma et al.^40^ showed that 78% of Peruvian adolescents living at sea-level do not meet the WHO recommendations for moderate-to-vigorous physical activity. In contrast, children and adolescents from the Amazon region tend to live in cities with less population density but larger areas. Peru’s sea-level region represents 11% of the total area of the country but it is occupied by 65% of the total population, while the Amazon region represents 60% of the total area but only 5% of the total population reside in this region (approximately 1.6 million)^41^. Moreover, most families in the Amazon region are dependent on agricultural production and children tend to assist their parents with these activities, especially outside of school hours. The time spent in agricultural pursuits may be providing these children with rich opportunities to play freely in the natural environments which in turn increases their physical activity and improves their GMC.

**Table 4:** Natural, socioeconomic, demographic, health care and cultural features of the three regions.

	Sea-level (Barranco)	Amazon region (Chanchamayo)	High-altitude (Junín)
**Natural characteristics**			
Altitude (m)	58	751	4107
Precipitation, and temperature (average)	Arid; semi-warm (18 °C)	Rainy; warm (24 °C)	Rainy; cold (12 °C)
Humidity	Humid	Very humid	Humid
**Socioeconomic characteristics**			
Human development index	0.72	0.52	0.44
Per capita family income	1440.6	785.1	512.7
Primary production	Trade/tourism	Agriculture/tourism	Stockbreeding/agriculture
**Demographic characteristics**			
Population (total)	8.564.867	411.011	1.272.890
Population density (people/km^2^)	236.6	10.2	27.7
**Basic access to health care**			
Health center	Yes	No	No
Public hospital	No	Yes	Yes
Private clinic	Yes	No	No
Hospital campaigns and tracking at school	No	Yes	Yes
**Infrastructure for physical activity and sports available**			
Parks	Yes	Yes	Yes
Playground	Yes	Yes	Yes
Pool	Yes	Yes	Yes
Multisport indoor	Yes	Yes	Yes
Multisport outdoor	No	Yes	Yes
Gymnastics complex	No	No	No

Finally, when considering main effects of other individual characteristics, our results are fairly consistent with previous research. We found that biological maturation (favoring those less advanced), nutritional status (favoring normal weight), stunting (favoring non-stunted) and physically fitness (favoring higher levels) were all negatively associated with below normal GMC scores^15, 27^. These results are important pointers regarding their adverse and/or protective effects of such characteristics on the development of below normal GMC scores. More specifically, nutritional status and stunting are risk factors while physical fitness is a protective factor in predicting below normal GMC scores. Moreover, these findings are concordant not only with other Peruvian studies^12, 27^ but also with data from Portuguese^16^ and Flemish^30, 42^ children and adolescents.

There is little doubt that the school environment is important when considering optimal physical growth, fundamental motor skills and GMC development^6^. Our findings showed that 15% of the total variance in GMC levels was accounted for by the school context. However, in the final model (Model 5) when we added school characteristics after adjusting for individual characteristics, natural environments and their interaction, this model did not fit the data better than the previous one (Model 4). This result suggests that individual characteristics and the natural environment play a more important roles than school characteristics in the prediction of GMC problems in these Peruvian children and adolescents. This reinforces our previous suggestion about the differences between regions being highly important in explaining differences between children and adolescents with below normal GMC scores. This reiterates the importance of population lifestyles and highlights the importance of further research between different countries and regions, as well as contrasting high-income versus low-income countries.

This study is not without limitations. Firstly, the cross-sectional design does not allow any causal interpretation into the dynamics of individual and environmental complex relationships on changes in below normal GMC scores development. Secondly, the unbalanced sample sizes between regions (19.8% sea-level, 25.2% high-altitude and 55% amazon) limits the generalization of the results to all Peruvian youth. Thirdly, we did not have any information on other behaviors that could have influenced the links between stunting and below normal GMC scores, such as lifestyle behaviors (e.g., physical activity). Finally, we did not have any information about aspects of the home environment that may have interacted with individual characteristics and their development, which, in turn, may have had a protective or negative effect on GMC problems.

In conclusion, we showed a high prevalence of below normal GMC scores in Peruvian children and adolescents which were more pronounced in girls, who were aged 11–14 years from sea-level regions. Further, these findings highlight the important influences of individual and environmental characteristics on below normal GMC scores. This finding has key implications for physical education teachers to promote adequate levels of GMC development, as well as for pediatric care within local health-systems. Altogether, these results revealed the need to offer distinct physical education programs according to regions (i.e., sea level, high-altitude and amazon region) to accommodate their dissimilar characteristics. Further, early identification of overweight/obesity status, stunting and physical fitness levels aligned with biological maturation may also help to implement precise intervention programs tailored to children and adolescents’ characteristics. If subjects differ in some characteristics that condition their GMC unfolding, then there is a need to develop suitable pediatric care to foster and enhance their healthy growth and proper motor development.

## Methods

### Participants and geographical area of residence

Participants in the study were drawn from *“The Peruvian Health and Optimist Growth Study”*, which was conducted between November 2009 and July 2010. This study investigated the relationships between physical growth, motor development and health in Peruvian children and adolescents and their families^43^. Participants were recruited from 18 randomly chosen schools from the 78 schools in these three regions, and the original sample consisted of 10,424 boys and girls 6–17 years of age. Complete data were obtained from 7401 participants (4121 girls; 3280 boys 6 to 14 years old)—the age range of the GMC test battery was from 5 to 14.99 years.

Given the country’s heterogeneity in geography, participants came from three distinct regions: sea-level, Amazon region and high-altitude. Barranco was the chosen city at sea-level in the Lima region. The cities of La Merced and San Ramon in the Chanchamayo district represented the Amazon region, and the Junín district was used to represent the high-altitude location. Participants included in the present study were natives of their respective regions (non-immigrants). Information with regards to birthplace and current place of residence was collected from individual’s identity cards. Table 4 shows the distinct characteristics of these geographical locations, based on information provided by National Institute of Statistics and Informatics (INEI)^44^, city-halls^45–47^ and the digital platform of the Ministry for the Environment^48^.

Written informed consent was obtained from legal guardians, and the project was approved by the local school and political authorities, as well as by the Ethics Committee of the National University of Education Enrique Guzmán y Valle (UNE EGyV). All methods were performed in accordance with the relevant guidelines and regulations. Moreover, the study was performed in accordance with the ethical standards established in the Declaration of Helsinki.

### Outcome variable

#### Gross motor coordination

Gross motor coordination was assessed using the *Körperkoordinationtest für Kinder* (KTK), developed by Kiphard and Schilling^49^ for children and adolescents aged 5–14.99 years of age. This test battery has systematically been used in European^50^, African^51^ and South-America populations^12, 52^. The KTK is explained in detail elsewhere^39^. In brief, the battery contains four tests: walking backwards along a balance beam, hopping on one foot, jumping sideways, and moving sideways on boxes. A total KTK score is obtained from summing the scores obtained from each test. This unweighted sum of the scores, adjusted for age and sex, is named as the motor quotient (MQ), and has the following categories: (i) not possible (MQ < 56); (ii) severe motor disorder (MQ 56–70); (iii) moderate motor disorder (MQ 71–85); (iv) normal (MQ 86–115); (v) good (MQ 116–130); (vi) high (MQ ≥ 131). For the present study, we only considered two broad categories: MQ > 85 as ‘normal’ GMC, and MQ ≤ 85 as ‘below normal’ GMC, as recommended by Schilling^53^. In our statistical models, normal GMC was used as the reference category. ANOVA-based intraclass correlation reliability estimates of children and adolescents GMC performance ranged from 0.78 in the moving sideways test to 0.92 in the walking backwards test.

### Exposure variables

#### Anthropometry

Body measurements were made according to standardized protocols^28^. Height and sitting height were measured using a portable stadiometer (Sanny, Model ES-2060) with the subject’s head positioned in the Frankfurt plane, to the nearest 0.1 cm. Body mass was measured to the nearest 0.1 kg using a digital scale (Pesacon, Model IP68). Technical error of measurement (intra-observer error) was 0.2 cm for height, 0.1 cm for sitting height, and 0.1 kg for body mass. BMI was calculated by dividing weight (kg) by height squared (m^2^).

### Stunting and body weight status

Stunting (height-for-age) and body weight status (BMI-for-age) were predicted using age- and sex-specific WHO Child Growth Standards^54, 55^. Two stunting groups were created: normal growth [height-for-age Z score ≥ − 2 standard deviation (SD)], and stunted growth (height-for-age Z score < − 2 SD). Normal growth was used as the reference category in the models. Three body weight status groups were created: thinness (BMI-for-age < − 2 SD), normal weight (BMI-for-age between ≥ − 2 SD to ≤ 1 SD), and overweight/obese (BMI-for-age > 1 SD). However, given that only 40 subjects were classified with thinness (0.5% of the sample, representing 25 females and 15 males, 2 from sea-level, 15 from high-altitude and 23 from Amazon region), in the final models only normal weight and overweight/obese groups were considered and a thinness/normal weight grouping was used as the reference category.

### Biological maturation

Biological maturation was assessed using a measure of somatic maturity predicted from anthropometrics to calculate a maturity offset value^56^. Maturity offset (years from peak height velocity) is an estimated temporal distance and is expressed in decimal years. Age at peak height velocity (PHV) is calculated as chronological age at assessment minus maturity offset. A positive (+) maturity offset indicates the number of years the participant is beyond attainment of PHV, whereas a negative (−) maturity offset represents the number of years the participant is before attaining PHV. This method has been widely used in children and adolescents^57–59^ and was previously used in other Peruvian studies^25, 60^.

### Physical fitness

Physical fitness was assessed using four tests: handgrip strength (static muscle strength component); standing long jump (explosive muscle strength component); shuttle-run (speed and agility component); and 12 min run (cardiorespiratory component). These tests are part of the EUROFIT test battery^61^ and the American Alliance for Health, Physical Education, Recreation and Dance (AAHPERD) test battery^62^. Reliability was estimated, and intraclass correlation values ranged from 0.79 in the shuttle-run test, to 0.85 in the handgrip test. Age-, and sex-standardized z-scores were computed for each test (the shuttle-run time was inverted), and then summed to obtain a total physical fitness z-score for each individual, as recommended^63, 64^.

### School characteristics

Information concerning school characteristics was obtained via a questionnaire completed by a school administrator, assisted by a research team member. A modified, and locally adapted, version of the healthy eating and physical activity modules of the healthy school planner designed by the Joint Consortium for School Health was used^65^. The questionnaire included information from five domains: school size and characterization (number of children, number of teachers and school setting); healthy eating and physical activity policies (the existence, or not of policies and practices used by the school board); extracurricular activities (the existence and type of extracurricular activities available in the school); frequency and duration of physical education classes; and school facilities (playground dimension and characterization, multi-sports roofed existence and dimension, number of structures and equipment available for physical education classes). For the present study, we only used information related to: school setting [mixed (reference category) or urban], number of students (continuous variable), policies and practices for physical activity [three categories: no policies nor practices (reference category); only policies; only practices], playground characteristics [with or without obstacles (reference category)], indoor multi-sport [existence or not (reference category)], physical education classes duration [two categories: < 90 min (reference category) and ≥ 90 min], active time during physical education classes (continuous variables), extracurricular activities [two categories: no (reference category) and yes] and availability of school infrastructures outside of school activities [two categories: no (reference category) and yes].

### Statistical analysis

Descriptive statistics are reported as means, standard deviations, and percentages as appropriate. Since the data were hierarchical in nature, i.e., participants nested within schools (two-levels), we used a multilevel logistic regression model (0 = normal GMC; 1 = below normal GMC scores) with a step-by-step modeling approach with increasing complexity: first, a null model with no predictors was estimated to calculate how much of the total variation in GMC categories was explained by the schools; second, five sequential models were built with varying complexity levels. Model 1 used age, sex and geographical area; Model 2 built on Model 1 and tested for two-way interactions: age-by-sex, age-by-geographical area and sex-by-geographical area; in Model 3 a three-way interaction was added, namely: age-by-sex-by-geographical area; Model 4 included other child-level characteristics such as stunting, body weight status, maturity offset and physical fitness; Model 5 included school-level characteristics. All model parameters were simultaneously estimated using maximum likelihood procedures, and when appropriate covariates were centered at their means as generally advocated^66^. Deviance (− 2 log likelihood value) was used as a relative measure of model fit, with smaller values indicating a better fit to the data. When comparing nested models, we relied on differences in deviance, which follows a chi-square distribution with degrees of freedom equal to the difference in the number of estimated parameters from both models. Stata 14 was used in all analyses, and the significance level was set at 5%.
